# Bacterial Cellulose-Based Blends and Composites: Versatile Biomaterials for Tissue Engineering Applications

**DOI:** 10.3390/ijms24020986

**Published:** 2023-01-04

**Authors:** Mahendra P. Raut, Emmanuel Asare, Syed Mohammad Daniel Syed Mohamed, Elliot N. Amadi, Ipsita Roy

**Affiliations:** Department of Materials Science and Engineering, Faculty of Engineering, University of Sheffield, Sheffield S3 7HQ, UK

**Keywords:** bacterial cellulose, BC composite/blend scaffolds, hard tissue engineering, soft tissue engineering

## Abstract

Cellulose of bacterial origin, known as bacterial cellulose (BC), is one of the most versatile biomaterials that has a huge potential in tissue engineering due to its favourable mechanical properties, high hydrophilicity, crystallinity, and purity. Additional properties such as porous nano-fibrillar 3D structure and a high degree of polymerisation of BC mimic the properties of the native extracellular matrix (ECM), making it an excellent material for the fabrication of composite scaffolds suitable for cell growth and tissue development. Recently, the fabrication of BC-based scaffolds, including composites and blends with nanomaterials, and other biocompatible polymers has received particular attention owing to their desirable properties for tissue engineering. These have proven to be promising advanced materials in hard and soft tissue engineering. This review presents the latest state-of-the-art modified/functionalised BC-based composites and blends as advanced materials in tissue engineering. Their applicability as an ideal biomaterial in targeted tissue repair including bone, cartilage, vascular, skin, nerve, and cardiac tissue has been discussed. Additionally, this review briefly summarises the latest updates on the production strategies and characterisation of BC and its composites and blends. Finally, the challenges in the future development and the direction of future research are also discussed.

## 1. Introduction

Tissue engineering is an interdisciplinary field that offers restoration, improvement, and replacement of damaged tissues aiming to re-establish the native functional properties of tissues using a combination of scaffolds, living cells, and growth factors [1]. Over the last two decades, the interest in tissue engineering for the regeneration of soft and hard tissues is increasing significantly because of the growing demand for successful tissue repair. As a result, the global tissue engineering market is projected to grow to USD 28.9 billion by 2027, expanding at a CAGR of 14.2% every year from 2020 to 2027 [2]. Since biomaterials are central in regenerative tissue engineering, the demand for biomaterials has also increased significantly in recent years.

Biomaterials allow the fabrication and design of scaffoldings that provide an appropriate natural physiological environment to support cell growth and tissue development by mimicking the properties of the natural extracellular matrix (ECM) [3]. Particularly, naturally derived biomaterials such as bacterial cellulose (BC), polyhydroxyalkanoates (PHAs), and alginate are gaining significant attention because they are remarkably biocompatible and biodegradable. Moreover, these biopolymers are sustainable materials because they can be produced using renewable resources, thus mitigating the need for fossil-based sources.

Within these, BC is a natural polysaccharide-based polymer produced by specialist acetic acid-producing bacterial genera to facilitate host-bacterial interaction [4] and/or as a protective biofilm envelope under harsh conditions in nature [5]. BC is a secreted product with unique structural and mechanical properties, produced via well-controlled biochemical pathways involving various catalytic enzymes [6]. For the last two decades, researchers have identified BC as an extremely beneficial biomaterial for biomedical engineering due to its remarkable properties such as its purity, water-holding capacity, biocompatibility, crystallinity, and porosity [7]. As a result, the global BC market has been steadily on the rise and is expected to grow from 250 million USD in 2019 to 680 million USD by 2025 [8].

However, due to its high polarity and strong intermolecular hydrogen bonding, BC has poor solubility in common solvents that hinder its use as a high-performance biomaterial in tissue engineering. Hence reinforcement of BC with materials of interest to produce modified BC composites/blends is imperative. Fortunately, the properties of BC can be significantly tailored by either controlling production methods (in situ) or by reinforcing BC with certain materials with desirable properties after production (ex situ) to produce suitable BC composites and blends with specific properties for wider applications in tissue engineering [7]. Recently, the combination of BC with nanomaterials and other biopolymers for the fabrication of BC-based scaffolds has received a lot of attention, and it has proven to be a promising advanced material in tissue engineering [9]. As a result, BC-based scaffolds have made significant progress in the past decade in the field of soft and hard tissue engineering. The properties of BC, such as the 3D porous structure with a large number of hydroxyl groups on the surface, facilitate electrostatic interactions and hydrogen bonding with nanoparticulate fillers and other polymers such as chitosan, dextran, glycerine, and PHAs. This has resulted in an enhanced mechanical property of the final material of interest [10,11,12,13,14,15,16,17,18,19,20].

**Figure 1:**
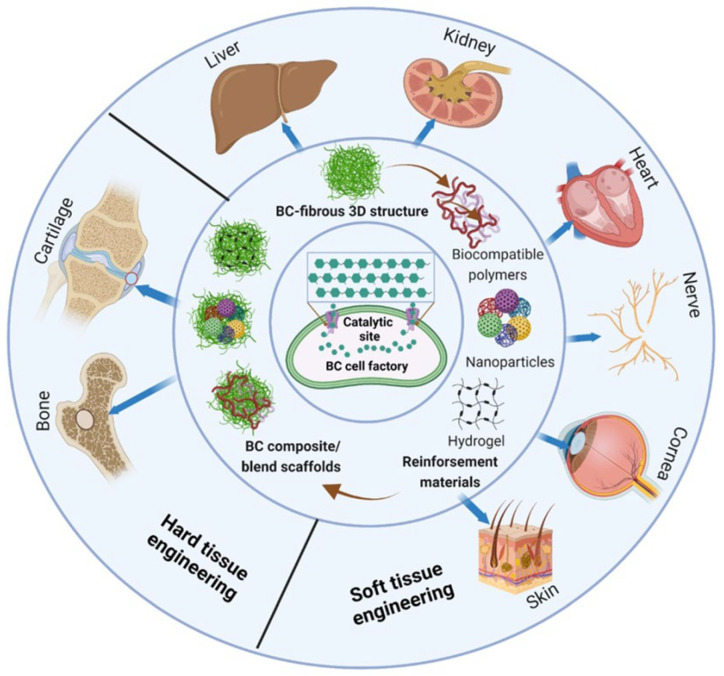
Schematic illustration of the application of BC-based composite/blend scaffolds in various regenerative tissue engineering (Created with BioRender.com; accessed on 28 November 2022).

In this review, we present the latest developments in designing of functionalised BC-based blend/composite scaffolds using various materials of interest (nanoparticles and other biopolymers) to be used as an advanced biomaterial for regenerative tissue engineering applications. As summarised in Figure 1, we will briefly present an update on biosynthesis, structural characteristics, and production of BC, followed by the current state of the art in research on the designing of functionalised BC scaffolds and their use in the repair and regeneration of various tissues including bone, cartilage, vascular, skin, nerve, and cardiac tissue. Finally, challenges in the future development and the direction of future research in BC scaffold-based tissue engineering are also discussed.

## 2. Biosynthesis, Structure and Characteristics of BC

### 2.1. Biosynthesis

**Figure 2:**
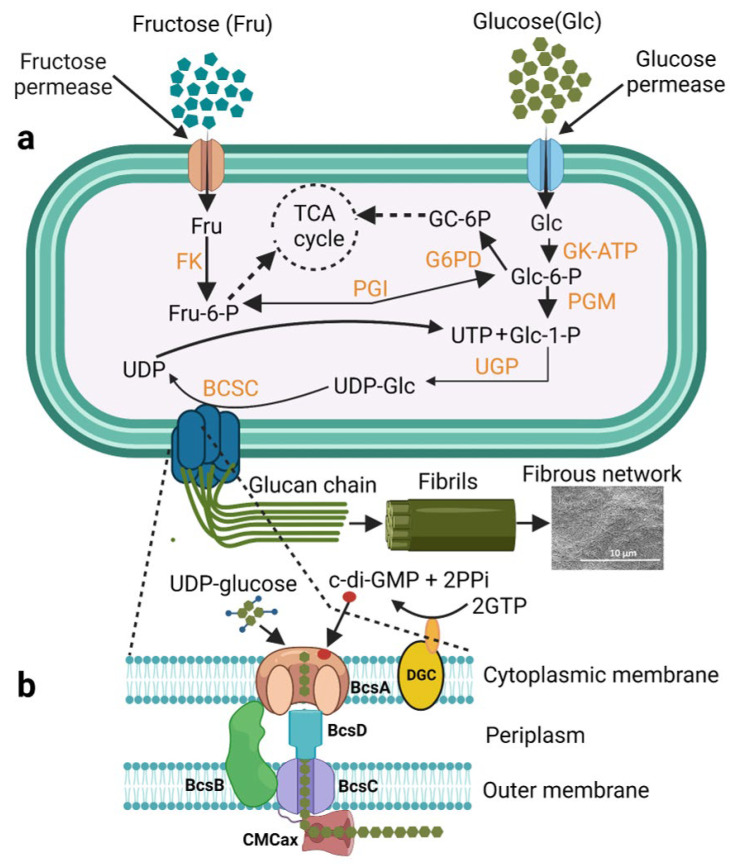
(**a**) The proposed mechanism of BC biosynthesis in *K. xylinus* using glucose and fructose as carbon sources and assembly of cellulose into nanofibrils. Glc; glucose, Glc-6-P; glucose 6 phosphate, Glc-1-P; glucose 1 phosphate, UDP-Glc; uridine diphosphoglucose (UDP-Glc), GC-6-P; gluconate 6 phosphate, Fru-6-P; fructose 6 phosphate, Fru; fructose, GK-ATP; ATP dependant glucokinase, PGM; Phosphoglucomutase, UGP; UDP–glucose pyrophosphorylase, G6PD; Glucose-6-phosphate dehydrogenase, PGI; phosphoglucoisomerase, FK; fructokinase and BCSC; bacterial cellulose synthase complex and (**b**) membrane-based cellulose synthase complex (Created with BioRender.com; access date 4 November 2022).

Plant-derived cellulose is one of the most abundant polymers on earth, being exploited for human benefit for thousands of years of civilisation. However, cellulose derived from bacteria has recently emerged as one of the preferred biomaterials among engineers and scientists due to its high suitability in the biomedical field. This is due to its remarkable properties such as purity, mechanical strength, water-holding capacity, porosity, crystallinity, and biocompatibility [21,22]. The known cellulose producers so far are the various genera of Gram-negative bacteria such as *Achromobacter*, *Pseudomonas*, *Acetobacter*, *Salmonella*, *Azotobacter*, and *Rhizobium* and the Gram-positive bacterium *Sarcina ventriculi*. Among all the above, Komagataeibacter (formerly known as Acetobacter and *Gluconacetobacter*) xylinus (*K. xylinus*) has been popularly used as a model organism to produce BC This is due to the fact that *K. xylinus* is a non-pathogenic bacterium and has been successfully used for the scale up BC production to the commercial level [5,22]. Therefore, in an effort to gain an in-depth understanding of the cellulose biosynthetic pathway and its regulation, *K. xylinus* was predominantly investigated. During BC biosynthesis, cellulose nanofibrils are secreted as an exopolysaccharide by the cells to the liquid and air interface. The BC biosynthesis, polymerisation, and translocation of microfibrils are carried out by a well-controlled membrane-associated multi-protein complex. The multi-protein complex consists of individual enzymes, catalytic subunits, and regulatory proteins. The most recent proposed model for BC biosynthesis consists of sequential biochemical reactions well orchestrated by four key enzymatic biocatalysts that include (1) glucokinase that catalyses the phosphorylation of glucose to glucose-6-P, (2) phosphoglucomutase that catalyses the isomerisation of glucose-6-P to glucose-1-P, (3) UDPG pyrophosphorylase (UGPase) that synthesises UDP-glucose (UDPGlc), and (4) finally the cellulose synthase complex (BcsA, BcsB, BcsC, and BcsD) that links two UDPGlc monomers during polymerisation [23,24]. It was found that the UDPG pyrophosphorylase was the key player in the overall biosynthetic process [25]. Additionally, cyclic-di-GMP (C-di-GMP), a biofilm-regulating secondary messenger was found to be a modulator for the cellulose synthase complex activity and regulated the BC biosynthesis by interacting with the protein BcsA and BcsB subunit via the *PilZ* domain of the complex in the periplasm [26,27]. In contrast, C-di-GMP was also seen to modulate the expression of the cellulose-degrading machinery in biofilm-producing anaerobes as reported by Raut et al. [28]. The BcsC and BcsD are outer-membrane subunits of the complex responsible for the assembly, crystallisation, and export of the synthesised cellulose chains [28]. Other proteins such as CcpAx, CMCax, and BlgAx are not directly involved in BC synthesis but play a vital role in the maintenance of the native structure of BC [29]. The proposed mechanism of cellulose biosynthesis in *K. xylinus* is shown in Figure 2.

### 2.2. Structure

BC has a chemical structure based on a 3D ultra-fine network of fibre structure, consisting of glucose monomers linked together by β-1 → 4 glycosidic bonds into the glucan chain. Parallel glucan chains then aggregate and are held together into protofibrils by hydrogen bonding. The protofibrils are secreted out through the cell wall and these further aggregate into nanofibrils and microfibril ribbons [30,31]. These ribbons create a web-shaped 3D network with abundant hydroxyl groups on the surface that provide unique material properties and strength to BC such as porosity, hydrophilicity, biodegradability, and the capacity for chemical modification [7,31]. The nano-porous structure, hydrophilic nature, and high surface-to-area ratio lead to the high water retention capacity of BC [31].

### 2.3. Crystallinity

The crystallinity of BC has been widely investigated. An extensive arrangement of the cellulosic microfibrils and ribbons forms a 3D structure that results in high crystallinity, which is key in forming pellicle sheets, especially in a static culture [32]. The crystallinity can be quantified by several analytical methods, such as X-ray diffraction (XRD), Infrared spectrum (IR), and Nuclear Magnetic Resonance (NMR). These analyses enable obtaining crystallinity index, which measures the crystallinity degree by percentage. The IR method evaluates the material’s ratio of crystalline to amorphous content in the cellulose [33].

Andritsou et al. [34] have used various analytical methods to compare the crystallinity of BC produced from citrus waste using *Komagataeibacter sucrofermentans* DSM 15973 with cellulose extracted from orange peel. Using XRD, the crystallinity was calculated using the peak intensity, as suggested by Segal [35]. The BC sheet produced was 87 ± 2% crystalline, which was much higher compared to the orange peel-extracted cellulose (50–60%). Additionally, the authors calculated the crystallinity index in terms of crystalline/amorphous ratio by IR analysis. The results showed that the ratio of crystalline to amorphous content was significantly higher in BC (9.7 ± 0.5) as compared to the orange peel cellulose (1.5–2.5) [34].

Drying is the BC processing method mainly used to remove its water content, which is another factor that influences the degree of crystallinity of BC [5]. The commonly used drying methods include air-, oven-, vacuum-, and freeze-drying. Oven-drying is widely reported to improve BC’s crystallinity and mechanical strength despite resulting in shrinkage, as suggested in several previous studies [36,37,38]. Another method to achieve a higher degree of crystallinity of BC is drying using supercritical CO_2_, compared to air- and freeze-dried samples [39]. However, a study by Muhammad et al. did not lead to any changes in crystallinity despite using the oven-, air-, and freeze-drying methods [40]. A similar observation was also noted by Vasconcellos and Farinas who showed around 85% degree of crystallinity for both methods when analysed by Fourier Transform Infrared spectroscopy [41]. Hence, BC crystallinity can be changed post-production, and it depends on drying methods used to obtain a water-free scaffold.

### 2.4. Solubility

BC is virtually insoluble in water and organic solvents. To facilitate certain applications of BC, it is the foremost requirement to dissolve BC.

Some organic solvents with specific salts can dissolve BC. For example, dimethylacetamide (DMAc) can dissolve BC in the presence of lithium chloride (LiCl), specifically at 0.4% *w*/*v*, with heating between 110 and 170 °C [42]. Shen et al. managed to dissolve BC in a lithium chloride/N,N-dimethylacetamide (LiCl/DMAc) solvent system at a maximum concentration of 3 wt% at 45 °C with sequential activation through the addition of KMnO_4._ [43]. Tilak et al. (2016) employed two solvent systems, trifluoroacetic acid (TFA) and dimethylsulphoxide (DMSO), at concentrations of BC from 2 to 5% under three conditions, including conventional heating at 60 °C, microwave, and cold treatment (0 °C). They concluded that microwave is a conducive technique to dissolve BC [44]. Another solvent system that has generally been used for cellulose dissolution is dimethyl sulfoxide/ammonium fluoride [45,46].

An aqueous system with certain salts also enables solubilisation of BC. Zinc chloride trihydrate, ZnCl_2_ 3H_2_O with a maximum 5.5 wt% concentration, has been used to dissolve BC, by heating at 80 °C [47]. Another aqueous system that is widely used is sodium hydroxide (NaOH), with varying urea concentrations, depending on the degree of BC polymerisation [48]. Recently, an ionic liquid, a salt in a liquid state has gained interest as a solvent system to dissolve BC such as 1-ethyl-3-methylimidazolium acetate [49] and 1-allyl-3-methylimidazolium chloride [50]. However, there are downsides to utilising the ionic liquid as a solvent. These chemicals are mostly not environment-friendly, highly toxic, and require post-treatment, such as dialysis, to remove the solvent component before utilisation, especially in tissue culture applications.

### 2.5. Biodegradability

Controllable biodegradation is a desirable feature of next-generation biomedical implants. Enzyme-aided hydrolysis is the main means by which cellulose becomes degraded by cellulose-degrading microorganisms. However, BC resists hydrolytic degradation due to its rigid structure. Moreover, the cellulose-degrading enzymes (cellulases) responsible for cellulose degradation by hydrolysing β-1,4 D-glucose linkages are lacking in the human body; therefore, BC is unable to degrade under the body’s physiological conditions. Some reports suggest that this could be an advantage since it provides a window for the degradation of BC to be tuned for specific applications [30]. A few studies have explored the functionalisation of BC either through in situ or ex situ means in order to render it degradable before in vivo applications. The common methods used to obtain biodegradable BC is through oxidation of the glucose units by using chemicals such as hypochlorite, nitrogen dioxide [51], periodate oxidation [52], and 2,2,6,6-tetramethylpiperidine-1-oxyradical (TEMPO) oxidation [53]. Oxidation is a preferred choice in many studies because it has proven effective and is believed to be a process that does not change the polymeric structure. Moreover, its interaction with the polymer is by either partial or full covalent bonds by converting the alcoholic groups to carbonyl or carboxyl groups. Other methods that have been linked to BC functionalisation with respect to their degradation include esterification, copolymerisation, etherification, crosslinking reaction, and grafting [54].

In their quest to improve the biocompatibility of BC and render it biodegradable, Favi et al. (2016) prepared microporous BC scaffolds using the laser patterning technique. The scaffolds were oxidised via periodate oxidation to make them biodegradable. Native BC is known to possess nanopores which tend to impede the ingrowth of cells, thereby limiting their full benefits to tissue engineering applications. To test the potential of the improved BC scaffolds in bone tissue engineering, the oxidised BC scaffolds were mineralised with nano-hydroxyapatite (nHA) to mimic the properties of the inorganic components of native bone tissues. These modified scaffolds were tested in vitro for their cytocompatibility with human-derived bone marrow mesenchymal stem cells (hMSCs) which comfortably adhered to and showed good viability on the BC composite scaffolds. Additionally, in vitro degradation studies with the scaffolds demonstrated a significant 13–15% loss in weight confirming that oxidation with periodate indeed conferred biodegradation properties on BC [52].

A recent study by Luz et al. (2020) claims that the degradation rate is directly related to the degree of oxidation which is a function of the reaction time. This is of particular interest in tissue engineering as different applications may require different rates of degradation. In addition, the study showed that oxidation influenced the swelling and crystallinity of BC [54]. Similarly, the authors demonstrated in another study that oxidised BC degraded effectively in in vivo conditions. Here, BC membranes were oxidised according to their protocol published in [54] and mineralised via immersion in strontium chloride solution and sodium dihydrogen phosphate. The results showed that oxidation enhanced degradation in vivo using the mice model [55].

### 2.6. Biocompatibility

Biocompatibility is another absolute requirement for the use of biomaterials in the clinic, which means it should not be toxic to biological systems [56]. Biomaterials have to meet the fundamental biocompatibility criteria set by the International Standards Organization (ISO 10993). They must not be toxic, thrombogenic, carcinogenic, antigenic, and mutagenic as specified in international standards [57]. Biocompatibility can be tested in in vitro and in vivo depending on the end-use application of the material. Various tests have been described in the recent review paper by Huzum et al. (2021), such as cytocompatibility tests, cytotoxicity elution test, MTT assay, agar overlay assay, genotoxicity evaluation, mouse lymphoma assay, in vitro chromosomal aberration assay, reverse mutation assay, irritation (intracutaneous reactivity) testing, skin sensitisation assay, acute systemic toxicity testing, haemocompatibility, coagulation, haematology testing, platelet activation testing, complement system activation, and implantation tests [56].

BC and BC-based scaffolds have been in use for various biomedical applications including tissue engineering and wound dressings since BC is proven to be biocompatible and nontoxic to the cells. In some cases, it is found that BC does not support cell adhesion which is an essential step in cell growth. For tissue engineering applications the material surface plays an important role in cell adhesion. Therefore, properties like wettability, porosity and surface chemistry of BC need to be improved for tissue engineering applications. Several approaches have been recently employed to improve the biocompatibility of BC such as modified BC via carboxymethylation [58], BC modified with chitosan [59,60], silk fibroin-modified BC (SF) [61], BC scaffold with nano bioactive glass [62], inorganic calcium filled BC hydrogel scaffold [63], and kanamycin grafted regenerated BC membrane [64].

## 3. Production of BC

**Figure 3:**
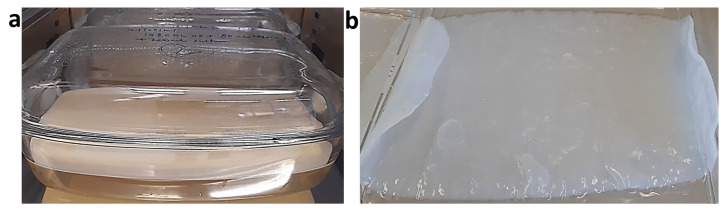
(**a**) BC produced under the static conditions in Professor Roy’s laboratory, University of Sheffield, using the bacterial strain *K. xylinus*, (**b**) Purified BC.

BC can be produced in static, agitated, or stirred conditions by the fermentation process. Different production conditions lead to different forms of cellulose. Under static condition, the BC yield depends on the concentration of the carbon substrate and the oxygen supply [31]. Usually, oxygen depletion and container size affect the overall productivity under static conditions. However, static production is simple and therefore still recommended for industrial-scale production. The major issues with static production are the requirements of high person power and large surface area for the scaled-up of the production. Figure 3 shows BC production under static condition in the laboratory of Professor I. Roy, University of Sheffield, Sheffield, UK.

Agitated and stirred conditions, on the other hand, produce BC with irregular morphology of fibrous suspension, spheres, pellets, or irregular masses and with lower crystallinity, mechanical strength, and degree of polymerisation [65]. This leads to inconsistent morphologies on the top and bottom layer of the produced BC [24,66]. Agitated conditions can also lead to the formation of cell mutants. Additionally, the quality and quantity of the final product is significantly affected by the quantity and quality of the inoculum. To combat these problems, novel bioreactors have been developed that could provide an alternate solution. Various bioreactors such as the stirred tank, airlift, rotating disk, aerosol, and membrane reactors have been designed to improve production efficiency of BC whilst maintaining its excellent properties [7]. Moreover, several efforts have been made to optimise growth conditions and the fermentation parameters such as culture media, carbon/nitrogen sources, oxygen, and pH to improve productivity and also to obtain modified BC properties during the fermentation [22].

Please replace highlighted sentence with corrected and revised sentence below “Recently, significant progress has been seen in BC production, and it has become a commercial reality in many industrial applications such as healthcare industry (wound dressings) (DermafillTM, Cellulose Solutions ltd, Colquitt, Georgia, Nanoderm™, Axcelon BioPolymers Corp, Ontario, Canada; CellulonTM, CP Kelco Atlanta, Georgia; and Gengiflex®®, Biofill Produtos Biotechnologicos ltd, Curitiba, PR, Brazil), food industry (Nata-de-coco, Profood International Corp, Philippine), and cosmetic industry (Nanomasque, GmbH, Germany) [7]. Owing to its remarkable mechanical properties, many researchers are now exploiting BC for even more sophisticated biomedical applications such as tissue engineering and regenerative medicine. However, BC production is expensive because of the low productivity (maximum BC yield so far reported is 20 g/L [67]) and the high production cost because of the use of pure sugars as a substrate. To keep BC production cost-effective whilst improving efficiency, the choice of cultivation conditions, selection of cheap substrate, and selection of robust bacterial strains are extremely important. Recently, Urbina et al., 2021 [68], Rathinamoorthy et al., 2020 [69], and Hussain et al., 2019 [70] have highlighted in their respective reviews about recent advancements in sustainable BC production using agro-industrial waste as substrates. Nonetheless, rigorous research is needed (1) to increase the yield, (2) to lower the production cost, and (3) to improve the commercial viability of BC. Moreover, in the last five years, reprogramming of the bacterial cells using genetic engineering, metabolic engineering, and synthetic biology have also contributed to improved BC production yields [71].

## 4. BC Blends and Composites

### 4.1. Production of BC-Based Blends and Composites and their Characterisation

To enhance the properties of BC and enhance its use as an advanced biomaterial for tissue engineering and regenerative medicine, unique structural features and mechanical properties must be introduced using the derivatisation of the BC scaffolds. Functionalised BC can be obtained via chemical modifications through derivatisation of the reactive hydroxyl groups on BC. Usually, based on the application, the material of interest can be incorporated into the BC structure during production (in situ) or after production (ex situ) to enhance its properties [7,72].

Although recent advancements in research have revealed BC as a versatile innovative biomaterial, the field of tissue engineering is yet to benefit satisfactorily from the full potential of BC [73]. This shortfall is mainly attributed to certain inherent drawbacks of BC such as irregular pore configuration and slow degradation or non-degradability, especially under physiological conditions [74]. Thus, the need to further modify and tune the properties of BC to meet the requirements of the application in question has attracted much attention.

The properties of BC can be significantly tailored by reinforcing BC with preferable materials to produce BC composites. In this context, both in situ and ex situ modifications are often used to alter the intrinsic chemical, physical, and mechanical properties of BC. Typically, post-production, purified BC can be obtained by removing bacterial cells and other extracellular components using an alkaline solution of sodium hydroxide at around 80 °C [75]. This purified BC can later be modified using several techniques to produce blends and composites of BC by reinforcing it with other beneficial materials. To ascertain functionalisation or modification of modified BC-scaffolds, several characterisation techniques are used such as Raman and Fourier transform infrared (FTIR) spectroscopy, scanning electron microscopy (SEM), field emission scanning electron microscopy (FESEM), X-ray diffraction (XRD), crystallography and surface analysis by profilometer spectroscopy, mechanical testing, and water absorption testing.

#### 4.1.1. In Situ Modification

The conventional practice of modifying BC after production often requires multi-step techniques and also requires certain compounds that are toxic that may cause many problems such as environmental pollution, low outcomes of reaction efficiency, and alteration of functional moieties [76]. Alternatively, in situ modification introduces desired changes to the nanofibrillar network of native BC during the bacterial fermentation; this serves as a better and more sustainable way of forming BC composites, while removing side effects associated with the post-production modifications. This is usually achieved by varying the growth conditions and by incorporating certain additives or reinforcing agents during fermentation in real time. In situ modification has been previously used to produce BC composites with unique and improved properties [73].

Dhar et al. [13] made a safer and sustainable composite of BC/graphene by incorporating sheets of reduced graphene oxide (RGO) into the BC membrane during in situ production. The results from the experiment indicated that not only were the fermentation kinetics improved at an optimal concentration of RGO (3 wt%), but also a strong intramolecular hydrogen bonding was established between the nanofibres of BC and partially oxidised RGO. The presence of such molecular interactions coupled with the hydrophobic property induced reduction of the RGO sheets. This led to the formation of a complex three-dimensional mesh-like network of BC nanofibrils that wrapped over RGO. The resultant BC/RGO matrix was notably flexible and exhibited a phenomenal electrical conductivity of 112 S/cm. This is also evident from its excellent mechanical attributes with a tensile strength of 151 MPa. This unique combination of desirable properties demonstrated the high prospects of BC/RGO nanocomposites in the fabrication of tissue engineering scaffolds with biosensing capabilities [13].

Similarly, a BC nanocomposite hydrogel was produced during an in situ production of BC using sodium alginate (SA) as an additive and tested for its potential in drug delivery systems [77]. The molecules of BC and SA interacted via hydrogen bonding resulting in the formation of a highly porous and entangled nanofibrous network. A range of varying concentrations comprising of different proportions of BC and SA were produced and tested for their thermal properties. The pore sizes were observed to be more regular and uniform with increasing SA content. However, it was confirmed that an SA content of 0.75% was the optimal concentration that achieved the enhanced thermal stability and dynamic swelling/de-swelling behaviour in the nanocomposite, as compared to BC alone, thus demonstrating suitability for drug delivery application. The in vitro assessment of the produced BC/SA nanocomposites showed that they were nontoxic to cells [77].

For its potential use as a stable antibacterial dressing, another study explored a unique in situ modification method of self-polymerisation of dopamine (PDA) to embed Tollen’s Reagent (Ag) into BC to form a BC/PDA/Ag nanocomposite which demonstrated good biocompatibility and excellent antimicrobial activity [78]. Zhou et al., performed in situ carboxymethylation of pristine BC using sodium carboxymethyl cellulose (CMC) as an additive, resulting in improved tensile strength and reduced elasticity of the fabricated BC scaffold, which significantly enhanced cell adhesion, proliferation, and biocompatibility in the context of an in vivo implantation [58].

#### 4.1.2. Ex Situ Modification

Forming a BC composite needs a particular treatment strategy, given its innate insolubility in virtually all organic solvents. One approach utilised lithium hydroxide/urea/thiourea aqueous system as a chemical treatment to solubilise BC before blending with alginate. Zhang and Luo [79] managed to break the hydrogen bonding within the BC in the study, which yielded a clear solution. They then mixed the BC solution with alginate before spinning using a wet-spinning apparatus to reproduce the fibres in a dilute sulphuric acid solution [79]. Characterisation of the fibres was carried out using FTIR, which revealed a significant shift of the carboxyl groups of alginate from 1622 cm^−1^ to 1608 cm^−1^, indicating hydrogen bonding with the hydroxyl groups within BC. The morphology of BC was changed with this blending approach, yielding larger circular pores, presumably due to the new crosslinking mechanism involving the interaction of alginate carboxyl groups with the exposed BC hydroxyl groups. Hence, the hypothesis suggested that alginate disturbs the hydrogen bond in BC by increasing the BC molecular motion and affects the final integrity of the blend [79].

**Figure 4:**
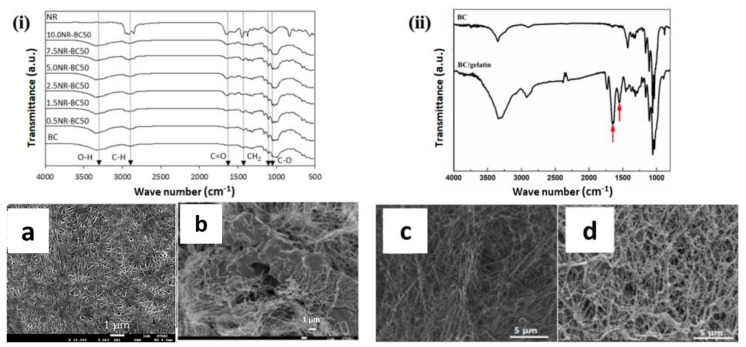
FTIR spectra and SEM micrographs; (**i**) FTIR of BC blended with natural rubber (NR) with varying NR composition, and FESEM micrograph for comparison (**a**) neat dried BC and (**b**) dried BC/NR blend, adapted from Potivara & Phisalapong [81]; and (**ii**) FTIR of BC/gelatin blend with neat BC and BC/gelatin blend (red arrows indicating signals from amide groups), with FESEM images comparison of (**c**) neat BC and (**d**) BC/gelatin blend, adapted from [82].

In an earlier study, Phisalaphong et al. [80] used a similar method in an aqueous system consisting of sodium hydroxide and urea before blending with alginate. Later, a BC/alginate blend film was formed by casting precipitation using 5 wt% calcium chloride solution, followed by treatment with 1% hydrochloric acid (HCl) solution before washing with distilled water. A FTIR analysis observed a shift of the alginate carboxyl group from 1598 cm^−1^ to 1602 cm^−1^–1616 cm^−1^, depending on the BC/alginate ratio, indicating specific interaction of the carboxyl group with the BC hydroxyl groups within the blend (Figure 4ii). However, the tensile strength of the BC/alginate blend decreased as the alginate content increased, with 3.38 MPa for 20% alginate content and 1.67 MPa for 80%, in comparison with 4.32 MPa for neat BC.

Meanwhile, BC has also been reinforced with natural rubber (NR) to yield a product with higher mechanical strength. Potivara and Phisalaphong optimised the ultimate tensile strength of BC from 112.4 MPa to 392.5 MPa, a four-fold increase, with a Young’s modulus value of 20.1 GPa. This blend included 2.5 wt% of NR and was prepared at 50 °C suspension temperature [81]. FTIR characterisation suggested that there were intermolecular interactions that reduced the fragility of BC. Both the BC and NR characteristic peaks were observed; BC was observed at 3347 cm^−1^ for stretching between O-H and C-H at 2900–2800 cm^−1^, meanwhile a high intensity -CH_2_ signal was observed at 1440 cm^−1^ and C = C stretching at 1637 cm^−1^ for NR. The physical property of the BC/NR blend was also different with more packed and dense morphology, compared to the mostly fibrous neat BC (Figure 4i(a,b)).

Wang et al. [82] have successfully made a composite hydrogel consisting of BC and gelatin to improve biocompatibility for the development of 3D tumour cell culture. The hydrogel was prepared by soaking the BC with aqueous gelatin solution to allow absorption and cross-linked with procyanidin solution. The BC/gelatin hydrogel scaffold showed obvious changes in terms of the presence of the amide groups in gelatin at 1650 cm^−1^ and 1540 cm^−1^, with a shift from 1670 cm^−1^ to 1650 cm^−1^, indicating prominent incorporation of gelatin molecules entwined within the BC matrix (Figure 4ii). The SEM image showed an extensive fibrous network, visible in the composite (Figure 4d). Similar to alginate, the presence of gelatin reduced the tensile strength of BC from 0.6 MPa for neat BC to 0.5 MPa and Young’s modulus of 11.8 MPa to 10.4 MPa for the BC/gelatin composite.

BC has also been physically treated using a blender or homogeniser to shred the pellicle into smaller bits and to form a slurry. Indrarti et al. [83] managed to physically blend BC with sorbitol, glycerol, and carboxymethyl cellulose (CMC). Even though this processing approach is simple, analyses showed significant changes in BC, especially for its physical properties. The extra components contributed as plasticisers, besides serving as cross-linkers. The tensile strength of BC, specifically blended with CMC, was significantly improved by more than two-fold. This was because the intramolecular interactions within BC were replaced by new interactions between the plasticiser and BC, involving mainly hydrogen bonds which reduced stiffness and promoted elasticity. This was evident from the FTIR characterisation which showed a slight difference across the samples, especially for a shift of carboxyl groups in BC/CMC composite at 1602 cm^−1^ from 1650 cm^−1^ in neat BC [83]. A conductive bacterial cellulose/polyaniline (BC/PANi) blends scaffold was successfully developed by polymerisation of aniline on to the BC network via interaction between CN and OH functional groups present on aniline and BC respectively [84]. This could be useful for biomedical applications.

#### 4.1.3. BC Modifications Using Nanofillers

In order to enhance BC properties, various kinds of nanofillers have been widely used in the fabrication of BC composites depending on the properties required for the application of interest. These include organic, inorganic, carbon nanostructures, and clay nanofillers [85]. Nanofiller reinforcement improves thermo-mechanical, electrical, and chemical properties without changing the density of the final product [86]. The use of various nanofillers in combination with BC is described in the following sections.

Chaabane et al. (2020) produced a novel magnetite nanomaterial containing complex [Fe_3_O_4_NP-INS-(DABC-EDA-Bzl)] using a multistep procedure. Briefly, 2,3-dialdehyde bacterial cellulose (DABC) was obtained by oxidation with sodium periodate (NaIO_4_) and then chemically modified with ethylenediamine (EDA) and benzil (Bzl) to produce (DABC-EDA-Bzl). [Fe(DABC-EDA-Bzl)Cl_2_] was prepared using Iron(II) chloride tetrahydrate (FeCl_2_.4H_2_O). Finally [Fe_3_O_4_NP-INS-(DABC-EDA-Bzl)] was obtained by in situ coprecipitation using Iron(III) chloride hexahydrate (FeCl_3_.6H_2_O) and ammonium hydroxide (NH_4_OH). This novel BC-based material exhibited excellent magnetic properties and had no cytotoxicity towards normal peripheral blood mononucleocyte (PBMC) cells and showed anti-tumour activity towards CT26 tumour cells in both in vitro and in vivo studies, offering an extraordinary platform in cancer chemotherapy [87]. In another study, a BC nanocomposite scaffold was fabricated using BC, magnetite, and hydroxyapatite using ultrasonic irradiation (BC-Fe_3_O_4_-HA). This exhibited excellent superparamagnetic characteristics and thermal properties, with biocompatibility towards human osteoblasts (MC3T3-E1 cell line) [88]. Bacterial cellulose nanocrystals (BCNCs) were produced by acid hydrolysis using hydrochloric acid (HCl) and sulphuric acid (H_2_SO_4_) of mechanically disrupted BC. The cationic surface modification of BCNCs was achieved via ionic interaction between surface sulphate groups of BCNCs and amines ((EDA, DM, MP, and AP) and amine-containing polymers (methacrylamide polymers; [_p_(DMAPMA·HCl_65_) and _p_(DMAPMA_65_-b-AEMA_76_)]). The resultant modified BCNCs scaffold showed good biocompatibility towards HeLa cells. In addition, cationic-modified BCNCs with methacrylamide polymers have shown potential as nucleic acid nanocarriers [89]. BC-based scaffolds with enhanced biodegradability were achieved by the oxidation process using sodium periodate, displaying high porosity with interconnected pores, with lower oxidation degrees, and suitable mechanical properties for peripheral nerve repair [51]. A thermally and electrically conductive biocompatible scaffold was fabricated using BC, polyaniline (PANI), and clay nanofillers by Salehi et al. [90]. The synthesised aerogel showed a high level of cell viability and no mutagenic activity towards L929 cells, confirming its potential application in tissue engineering applications. Barbi et al. successfully immobilised TiO_2_ and inorganic ceramic clay into BC, separately resulting in a hydrophilic porous membrane structure with clay and a plastic-like film with TiO_2_, suggesting potential applications in membranes for medical and textile related products, respectively [91]. Horue et al. [92] incorporated silver montmorillonite (MMT-Ag) with BC and showed excellent antimicrobial properties against Gram-positive and Gram-negative microbes, good biocompatibility towards fibroblast L929 cells, and a water holding capacity, suggesting favourable properties for wound healing applications. In a recent study, BC and halloysite nanotubes (HNTs) as a reinforcement incorporated into sodium caseinate (SC) improved mechanical, thermal, and barrier properties and also showed good biocompatibility against normal human fibroblasts, therefore offering potential future applications in food packaging and wound healing [93]. BC and the palygorskite clay (BC/PLG) based nanocomposite, loaded with metronidazole (MTZ), were able to control the water vapour permeation, offering suitable barrier properties for controlled drug release mechanism [94]. Hybrid composites of BC loaded with calcium phosphate (CP) were prepared by deposition by successive immersion in solutions of Ca(NO_3_)_2_·4H_2_O and (NH_4_)_2_HPO_4_, under ultrasonication. This BC/CP composite exhibited intrinsic magnetic properties that can be an excellent property enhancing cell attachment and growth, representing a promising candidate as a cement filler or for bone tissue engineering [95]. Recently Kim et al. (2022) produced a tough and stretchable 3D cryogel with enhanced mechanical strength using BC and poly(vinyl alcohol) (BC/PVA) by using the freeze-thawing process. The resultant scaffold showed good biocompatibility towards NIH 3T3 cells, therefore exhibiting potential for biomedical applications [96].

In a recent study by Wasim et al. (2022), a BC bioscaffold loaded with curcumin and montmorillonite (MMT) was prepared using dip coating and freeze-drying process. The combined effect of curcumin and MMT with BC showed ultraviolet-resistant properties and outstanding antistatic properties which can be used in many biomedical and food packaging applications [97]. Maruthupandy et al. prepared a highly antimicrobial photocatalyst to degrade organic dyes using BC, graphene, and magnetite (Fe_3_O_4_) (BC/Gr/Fe_3_O_4_) [98].

Besides the biomedical and tissue engineering applications, several BC scaffolds with nanofillers were developed with remarkable properties suitable for other applications. For instance, BC/starch/chitosan scaffold [99] and BC/lactic acid oligomer/poly lactic acid (PLA/OLLA-g-BC) were developed as green bioplastics for food packaging [100]. The biocomposite of BC/maple leaf fibres were developed as a leather substitute [101], BC/bentonite inorganic gel (BIG) clay/acrylic acid (AA) monomers were developed as superabsorbents [102]. Additionally, BC/polygonal magnetite nanoparticles (BC/MNPs) [103], BC/Ca-montmorillonite (Ca-MMT) [104], and BC/polyvinyl alcohol/graphene oxide/attapulgite (BC/PVA/GO/APT) were developed for wastewater treatment [105]. BC scaffolds with various fillers were also synthesised for electronic devices such as BC/Zn^2+-^ modified porous clay (cetrimonium chloride) [106], BC/halloysite nanotubes HNTs (HNTs-BC) [107], polyether block amide (PEBAX)/BC nanocrystals (BCNCs) [108], poly(vinyl alcohol) (PVA)/BC nanocrystals (BCNCs)/magnetite nanofiller (Fe_3_O_4_) (PVA/BCNC-Fe_3_O_4_) [109], BC/graphene/carbon nanotube/polyurethane [110], BC/graphene (GE) [111], BC/carbon nanotubes (CNTs) (BC-CNTs) [112], BC/2D titanium carbide (Ti_3_C_2_T_x_) nanosheets (also known as MXene) [113], and BC/platinum (Pt)/ruthenium (Ru)/and multiwalled carbon nanotubes (MWCNT) [114].

## 5. Application of Blends and Composites of BC in Tissue Engineering

The following sections will highlight the recent research status of the use of BC scaffolds in various tissue engineering applications.

### 5.1. Hard Tissue Engineering

#### 5.1.1. Bone

**Figure 5:**
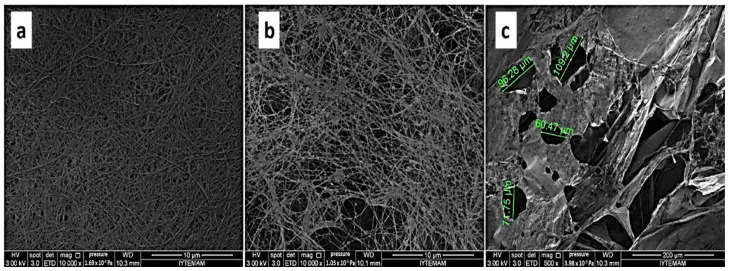
SEM images of the morphology of HAp-BC composites, produced for bone tissue engineering. (**a**) control (pure BC) at ×10,000 magnification, (**b**) HAp-BC composites at ×10,000 magnification, and (**c**) HAp-BC composites at ×5000 magnification (adapted from Bayir et al. [121]).

Bone is a heterogeneous composite material that employs an extracellular matrix (ECM) consisting of hydroxyapatite (HA) crystals in a collagen matrix and also contains minerals (mainly calcium phosphate), water, inorganic salts, and organic components (including collagen I) [115,116]. Trabecular bone is responsible for supporting movement in limbs and joints, whilst cortical bone provides mechanical support and protection [117]. Bone damage is common in conditions such as trauma, scoliosis, musculoskeletal pathologies (e.g., bone infection, tumours and osteoporosis), and diseases (e.g., osteomyelitis, osteitis, and osteoarthritis); all are the current clinical problems [118,119]. Allografts, autografts, xenografts, and other substitutes are already being employed to restore bone function in these cases [52,117]. However, these substitutes are not always suitable because of several limitations such as shortages of donor bone, sophisticated surgical procedures, disease transmission risk, death of grafted tissue, and host immune responses [120]. Scaffold-based bone tissue engineering strategies are now becoming more popular. The aim of bone tissue engineering is to regenerate bone via the combination of biomaterials, cells, and bioactive molecules in a three-dimensional (3D) matrix to provide therapy, creating a substitute that can repair and regenerate damaged tissue and also restrict disease transmission [120]. An example of BC scaffolds produced for bone tissue engineering purpose are shown in Figure 5. Table 1 shows proposed/potential BC-based composites tested for bone tissue engineering in recent studies.

Cao et al. developed a nanocomposite scaffold using an oxidised BC reinforced with Chitosan (CS) and nano-hydroxyapatite (nHA). The resultant scaffold showed improved mechanical properties, degradation rate, and water holding capacity as compared to a scaffold with only CS/nHA. The scaffold also showed good biocompatibility and improved cell proliferation when tested with MC3T3-E1 cells (a clonal murine cell line of immature osteoblasts derived from mice). In vivo study on a rat skull defect model provided evidence that this scaffold could induce bone tissue formation [127]. Zhu et al. [128] were also able to produce an effective BC composite scaffold reinforced with CS and alginate (Alg). This scaffold induced a tight fibre network structure and demonstrated effective swelling behaviour and compatible degradation rates. This composite exhibited good apatite formation, cytocompatibility, protein absorption, and release performance, therefore exhibiting great potential for BC-derived nanocomposites in bone tissue engineering applications.

A further study by Zha et al. [129] produced phosphorylated cellulose/sodium alginate (SA) sponge as a scaffold using a freeze-drying method. The inclusion of SA was able to improve the macroporous structure of the scaffold, and the phosphate groups grafted to the surface of BC were able to assist the formation of apatite crystals. The phosphorylated BC/SA composite exhibited good biocompatibility with L292 cells as compared to BC/SA composite and therefore proving its potential as a bone repair material.

Li et al. [130] fabricated an alginate/bacterial cellulose nanocrystals/chitosan/gelatin (Alg/BCNs/CS/GT) composite scaffold. This scaffold was observed to have a regular 3D morphology with a well-developed pore structure. The presence of BCN was able to control the swelling and biodegradability of the composite by embedding it into the alginate matrix via intermolecular hydrogen bonding. The scaffold structure promoted adhesion, proliferation, and spreading of the MG63 cells. The 3D morphology and well-developed pore structure, with controlled swelling and degradation behaviour, exhibited great potential for bone tissue engineering [130].

Recently, Khan et al. [131] reinforced BNC/β-glucan composite scaffolds with nano-hydroxyapatite (nHAp) and graphene oxide (GO) using acrylic acid monomer free radical polymerisation and a freeze-drying method. The structural and mechanical analysis of the resultant scaffolds showed great stability, spongy microstructure, porosity, and degradation properties. Cytocompatibility studies using MC3T3-E1 cells showed that this BC-composite supported the growth of the MC3T3-E1 cells due to its surface roughness, controlled porosity, improved mechanical properties, and significant biochemical affinity for cell adhesion and proliferation [131]. Another study by Khan and his co-workers [132] synthesised a porous scaffold of arabinoxylan (ARX), β-glucan (BG), nano-hydroxyapatite (nHAp), graphene oxide (GO), and acrylic acid (AAc) through free radical polymerisation and using the freeze-drying technique. The resulting scaffold possessed desirable morphological and structural properties in addition to the swelling, degradation, and mechanical behaviour. These structures were also found to be highly cytocompatible with MC3T3-E1 cell lines.

Recently Dubey et al. [133] observed that low dose BMP-2 primed murine mesenchymal stem cells (C3H10T1/2 cells) showed enhanced cell adhesion, cell growth, bone matrix secretion, osteoinduction, and maturation when seeded on 3D macro-microporous nanofibrous BC scaffold (mNBC) as compared to the un-primed cells. The authors suggested that further studies at the molecular level are needed to elucidate the underlying mechanism and regulatory pathways to corroborate these findings.

Further, researchers also fabricated porous composite scaffolds using silk fibroin and cellulose by dissolving in N, N-dimethylacetamide/LiCl solution and porous structure achieved using NaCl powder, and subsequently a hydrogel was prepared. In vitro study on MC3T3 osteoblast cells revealed that the resultant hydrogel supports differentiation of cells thus concluded to be a promising scaffold for bone tissue engineering [134]. Recently Barbosa et al. [135] developed scaffolds by 3D printing using mixtures of poly(ε-caprolactone) (PCL) and 45S5 Bioglass^®®^, labeled as synthesised bioglass (SBG). The scaffolds achieved a compression modulus range from 54.4 ± 14.2 to 155.9 ± 20.4 MPa, which is within the range of compression modulus required for bone tissue engineering. Cytotoxicity assays demonstrated non-toxic effects and cell viability for MG-63 cell proliferation and calcium deposition in all manufactured scaffolds.

#### 5.1.2. Cartilage

Cartilage is found in many areas of the body; it is a connective and flexible tissue which has many functions including absorbing shock, providing structural support, and supplying frictionless movement [136,137]. It is categorised into three types: hyaline, elastic, and fibrocartilage [136,137,138]. Cartilage has an extracellular matrix structure, made from ~80% water, collagen II, chondrocytes, and proteoglycans. Chondrocytes are the only cell type present in cartilage and constitute between 1 and 5% of the cartilage structure, and these cells are responsible for producing new cartilage post damage [139,140]. Cartilage damage can occur from injury, ageing, developmental disorders, and trauma. This damage can lead to osteoarthritis [139]. However, as cartilage is devoid of blood vessels, lymphatic connections, and nerves, chondrocytes struggle to regenerate and produce new cartilage [136,140]. There are three main procedures used to repair damaged cartilage; these include direct chondrocyte transplantation, bone marrow stimulation, and cell-culture treatment. Unfortunately, most treatment methods result in limited relief. This is a concern for long-term patients who may need to undergo many operations of limited effectiveness.

**Figure 6:**
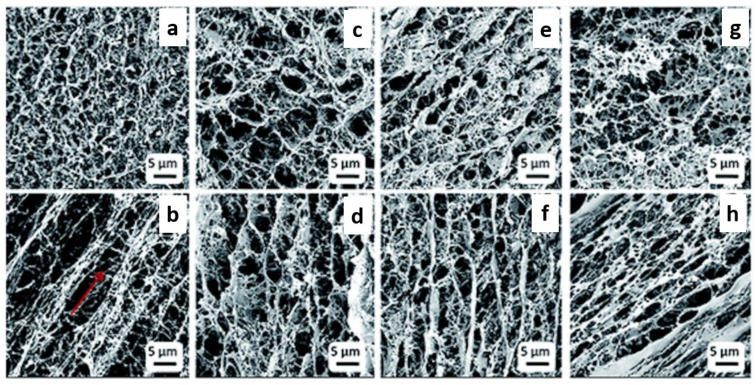
SEM images showing different morphologies of freeze-dried pure BC and BC/Chitosan (BC/Ch) composites obtained by varying the chitosan content. Pure BC (**a**,**b**); BC/Ch-1% (**c**,**d**); BC/Ch-1.5% (**e**,**f**); BC/Ch-2% (**g**,**h**). All images were taken at 5000× magnification with the top row showing the cross section and the bottom row is the inner wall of the composites (Adapted from Li et al. [141]).

Existing treatments for cartilage defects are very invasive, and therefore cartilage is the main target for tissue engineering [139]. Current treatments have been restricted through donor site morbidity, immunological rejection, scarce resources, pathogen transmission, and a lack of long-term solutions [139]. Therefore, tissue engineering has been looked at to grow new chondrocytes on an artificial scaffold where they can proliferate and regenerate. Similar to bone tissue engineering the choice of the scaffold is vital to regain normal functioning of damaged tissues and their regeneration. There were several BC-based composites synthesised recently that could be used as a cartilage repair material in clinical applications. Li et al. [141] incorporated chitosan into the BC network by using the freeze-drying method, increased the structural integrity, displayed excellent shape recovery, and exhibited mechanical properties and high porosity that were similar to the native human cartilage. Figure 6 shows SEM images of the high porosity of the BC/chitosan composites.

Li et al. [142] fabricated a 3D hierarchical porous BC/decellularised cartilage extracellular matrix (DCECM) scaffold using a freeze-drying technique after chemical crosslinking with N-hydroxysuccinimide (NHS)/N-[3-(dimethylamino)propyl]-N′-ethyl carbodiimide hydrochloride (EDC). This scaffold showed excellent cell adhesion and proliferation of chondrocyte cells derived from rabbits. In vivo study on this scaffold using rabbit cartilage defect model enhanced cartilage repair by regenerating tissues at cartilage defect site was seen as compared to native BC. The strong hydrophilicity and water retention ability gave this composite great elasticity and shape-memory properties in the wet state which is similar to the properties of native cartilage. Xun et al. [143] used N-hydroxysuccinimide (NHS)/N-[3-(dimethylamino)propyl]-N′-ethyl carbodiimide hydrochloride (EDC) to induce cross-linking on BC nanofibres. The resultant BC scaffold exhibited excellent compression properties and shape recovery ability. The scaffold also showed improved in vitro biocompatibility towards chondrocytes, compared to the pristine BC scaffold. In vivo studies on nude mice also demonstrated that this composite showed great biocompatibility and excellent ability to regenerate cartilage tissue. Akaraonye et al. (2016), developed poly(3-hydroxybutyrate)/microfibrillated bacterial cellulose (P(3HB)/MFBC) composites as 3D scaffolds for cartilage tissue engineering applications. This supported higher proliferation towards cartilaginous murine ATDC5 cells than the neat P(3HB) scaffold [136].

Further, Gu et al. synthesised BC/methacrylated gelatin (GelMA) composite hydrogels of high porosity and well-interconnected structures. The addition of methacrylated gelatin into BC was shown to improve mechanical properties of the scaffold. Increased GelMA content resulted in decreased pore size of the scaffold, suitable for cartilage tissue engineering applications. The chondrocytes encapsulated in GelMA/BC hydrogels were shown to support cell proliferation, whilst maintaining the correct chondrocytic phenotype, therefore showing potential for cartilage tissue engineering [144].

In another study by Wang et al., bacterial cellulose/silk fibroin double-network hydrogel was successfully prepared using aqueous silk fibroin (SF) solution as a modifier, without the use of any crosslinking agent. The resultant BC/SF scaffold showed an open porous and well-interconnected structure. The improved biocompatibility of the BC/SF scaffold towards preosteoblast cells (MC3T3-E1) was observed when compared to pure BC. Therefore, the properties exhibited by BC/SF double-network hydrogel were more suitable for cartilage tissue engineering as compared to neat BC [61].

### 5.2. Soft Tissue Engineering

#### 5.2.1. Kidney

In relation to kidney tissue engineering, limited studies were reported so far regarding the utilisation of BC as a potential material and scaffold for artificial kidney development. Nevertheless, the potential of BC in this area has been confirmed based on an assessment of its biocompatibility towards kidney cells as described below.

An established kidney cell line, Human Embryonic Kidney 293 cells (HEK 293) was used in testing the biocompatibility of a BC nanocomposite with a calcium-deficient hydroxyapatite powder by Grande et al. [145]. The process of incorporating the hydroxyapatite nanoparticle into BC was conducted in situ by adding the hydroxyapatite nanoparticle into the culture media prior to inoculation of *Gluconacetobacter saccharivorans* LMG 1582. The BC sheet for cell culture was produced by hot-press drying method at 105 °C, which involved pure BC and BC/carboxymethyl cellulose/hydroxyapatite or BC/CMC/HA composite [145]. CMC was an additive to increase the culture media viscosity to avoid the precipitation of hydroxyapatite. The BC/CMC/HA composite exhibited significant biocompatibility with the HEK 293 cells compared to that of tissue culture plastic (TCP). Even though the cells were not sticking to the material, BC was still deemed as biocompatible since the direct culture viability test revealed a viability up to 97.2% and hence confirming that this material could serve as a potential tissue engineering material. A further strategy, such as surface activation by plasma technology, will be required to improve cell adhesion properties.

It is worth mentioning that instead of BC, several studies report the usage of cellulose from plant origin as the filtration membrane material for the dialyser, in the form of regenerated cellulose (i.e., dissolved and re-precipitated cellulose). However, based on a couple of reports by MacLeod et al., cellulose as a dialysis membrane exhibits less biocompatibility and more immune response compared to synthetic membranes such as polysulfone [146]. On the contrary, cellulose triacetate [147] or cellulose diacetate [148] have been proven to have less platelet activation properties similar to polysulfone. Even though cellulose has mainly been used as a material to develop a membrane in the context of a damaged kidney, it is envisaged that the biocompatibility of this material may open up other potential applications in the kidney tissue engineering approach.

#### 5.2.2. Neural

Nerve regeneration is a complicated biological process due to the complex nature of the nervous system and its highly evolved processes. Unlike the central nervous system, peripheral nerves can self-regenerate when the injury gap is less than 5–10 mm [149]. However, injuries beyond this length regenerate poorly, posing a remarkable challenge in nerve tissue engineering. Generally, scaffold-based tissue regeneration depends largely on materials that are biocompatible, bioresorbable, flexible, semipermeable, and easily processable. However, a material with the ability to conduct applied electrical input is of great importance in regenerating nerve tissues [51,150]. Hence, to meet these requirements, materials that are electroactive are preferred [150]. Some studies have reinforced electrical functionalisation into BC by coating some conductive materials like multi-walled carbon nanotubes on BC scaffolds and by incorporating compounds such as graphene into the BC membrane. This has been proposed in several studies as a viable means of conferring electrical properties to BC [13,150,151]. Liu et al. investigated the effectiveness of nerve conduits made using a composite of BC membranes, poly(3,4-ethylenedioxythiophene), and sulphonated nanofibres (BPS) in a 12-week in vivo experiment. The electrically functionalised nanocomposite was prepared via the in situ polymerisation of poly(3,4-ethylenedioxythiophene) (PEDOT) into the BC wafers and doping it further with sulphonated nanofibres (SNFs). PEDOT was believed to have introduced conductive properties in BC which were further enhanced by the SNFs. The conductive nanocomposite exhibited high mechanical properties. Additionally, because of the self-assembled electrical conductivity exhibited on the BPS membrane, its surface roughness was improved, enhancing cell to cell communication, cell adhesion, and proliferation in the case of in vitro experimentation with adipose-derived stem cells (ADCSs). In comparison with hollow nerve tubes, nerve conduits made from the BPS composite exhibited superior regeneration capability of the rat sciatic nerve [152]. Another study led to the production of a pure natural BC hydrogel fibre (BCHF) using the wet-spinning method without the need of a crosslinker. The hydrogel fibres maintained their original high tensile strength and good ionic conductivity which demonstrated the potential of this strategy in making a next-generation neural interface [153]. Similarly, Yang et al. [154] developed a super soft BC-based neural interface as a better alternative to the existing ones that are mostly made from rigid and dense inorganic sources which tend to cause damage to tissues. The multichannel BC microarray was prepared by depositing layers of gold on thin BC (Au-BC). The recorded in vivo electrical activity together with their excellent mechanical properties which were commiserated with those of native tissues indicate that Au-BC electrodes have high potential in neural interfacing [154].

**Figure 7:**
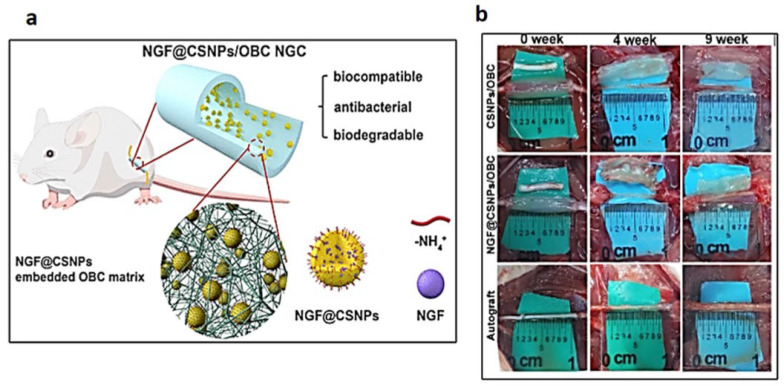
(**a**) Schematic representation of the process of incorporating growth factors into BC-based NGC and its implantation into a rat. (**b**) The digital photographs of the peripheral nerve regeneration, the BC conduits and transplantation at week 0, rat’s sciatic nerve regeneration at weeks 4 and 9 weeks post-surgery. Adapted from Wei et al. [156].

Furthermore, the addition of neurotrophic factors and cell therapies to a three-dimensional biomaterial-based nerve conduit has been proposed as better biomimetic devices to create the required microenvironment for optimal nerve regeneration. Cellulose-based biomaterials have been reputed for their promise as ideal drug carriers owing to their excellent biocompatibility, highly porous structures, tuneable stability, and mechanical properties [150,155]. To this end, Wei et al. [156] developed a novel in situ method of constructing a nerve growth factor (NGF) based BC nerve guide conduit by incorporating chitosan (CS) nanoparticles (CSNPs) encapsulated with NGF into an oxidised BC (OBC) conduit via the ion gel strategy (Figure 7). The solution of CS/NGF was added under controlled pressure into the OBC conduit to ensure a controlled release of the NGF. The NGF/CSNPs/OBC nanocomposite preserved its 3D network and mechanical properties. The conduit was found to be biodegradable, exhibiting ECM-like porous structure and antimicrobial resistance. The in vivo results showed that these novel conduits were comparable with autografts and, therefore, a promising ideal candidate for peripheral nerve regeneration.

Similarly, Robbins et al. [157] incorporated growth factors that were bonded covalently to the surface of BC via salinisation. Human embryonic stem cell-derived progenitor cells were cultured on the functionalised BC which supported the improved growth and differentiation of the stem cells into dopaminergic neuronal progenitors. The authors demonstrated this strategy as an effective means of utilising therapeutic cells to heal and restore functionality to damaged nerves in both the central and peripheral nervous systems [157].

To investigate BC as a new material that can easily be converted into tubes for the improvement of facial nerve regeneration, Binnetoglu et al. [158] fabricated a BC-based nerve conduit and performed an in vivo study using the facial nerve model of female Sprague Dawley rats. The results showed that the numbers of regenerating myelinated fibres increased significantly when BC conduits were used [158].

#### 5.2.3. Cardiovascular

Vascular diseases, myocardial infarction (MI), stroke, etc. are common cardiovascular ailments that account for most of the causes of deaths across the globe. MI commonly results in cell necrosis around areas of the heart which leads to poor functioning of the heart. The search for suitable biological scaffolds for the repair of infarcted myocardium has been on the rise in recent years, and results thus far have confirmed their enormous support to the regeneration of injured tissues [159]. Therefore, biological scaffolds such as stents and vascular grafts may be indispensable approaches in the treatment of cardiovascular diseases. However, one of the daunting challenges in tissue engineering is the replacement of blood vessels. This is because the organs in the human body are made up of complex internal and highly branching vascular systems that carry blood containing nutrients and oxygen to and from the heart. Hence, fabricating a biomimetic vascular graft that provides a long range functionality cannot be underestimated from both material and manufacturing perspectives [160]. The lack of an appropriate material is largely attributed to the poor functional outcomes of the current available devices. Nevertheless, bioresorbable and biocompatible nanomaterials have been presented as highly promising materials [160]. Studies have confirmed improvements in mechanical and surface properties, haemocompatibility, and antithrombogenicity when nanocomposite materials were incorporated into stents and cardiovascular grafts [161]. BC nanocomposites present a unique set of favourable characteristics, justifying the recent surge in their application in cardiac tissue engineering [161]. Hobzova et al. [20] embedded poly(2-hydroxyethyl methacrylate) (PHEMA) into the BC nanofibrous network via in situ UV radical polymerisation of the monomers of PHEMA into the nanofibrous structure of BC. Results showed significant enhancement of the mechanical and biocompatibility properties of BC/PHEMA nanocomposite [20]. Basnett et al. developed novel 2D poly(3-hydroxyoctanoate)/bacterial cellulose (P(3HO)/BC) composites with enhanced degradability compared to neat P(3HO) and demonstrated increased cell growth and proliferation for human microvascular endothelial cell line (HMEC-1), confirming potential applications in the development of biodegradable stents [162].

Another key consideration in tissue engineering is the possibility of using advanced technologies such as 3D bioprinting to fabricate constructs that closely match native tissues and organs. In this regard, Lei et al. [163] demonstrated the superiority of using scalable 3D printing techniques to create scaffolds that mimic natural vascular networks. Their perfusable and permeable hierarchical microchannel networks (PHMs) produced via novel 3D printing technology showed a complex 3D framework with varying length scales and structurally organised features. This created a suitable ECM environment where the interconnected microchannels along with its controllable microporous walls allowed the exchange of essential nutrients and metabolic products which showed that the PHM-based cardiac patch could significantly reduce fibrosis following myocardial infarction [163].

Thanks to its nanofibrillar network, BC can connect with a variety of polymers to form nanocomposites that possess desirable characteristics and processable qualities pertaining to the demands of cardiovascular TE [160]. Ma et al. [164] fabricated a chain of photocrosslinkable composite hydrogels mNCC-MeGel (mNG) by conjugating TEMPO-modified nanocrystalline cellulose (mNCC) onto the backbone of methacrylated gelatin (MeGel). The mNCC-MeGel (mNG) nanocomposite displayed enhanced mechanical properties. To prove the concept, the mNG hydrogel was combined with a viscosity enhancing agent and used to 3D bioprint a tall, self-supporting tubular construct which showed good cell viability after 7 days [164].

In an attempt to facilitate cardiac conduction across a disrupted myocardium, Pedrotty et al. [165] developed a stretchable, flexible, and conductive biopatch by 3D printing carbon nanotube ink on BC. In vivo studies confirmed that the patch successfully restored conduction across the affected area and hence was a viable means of restoring electrical conduction in the abnormal areas of the heart [165].

**Figure 8:**
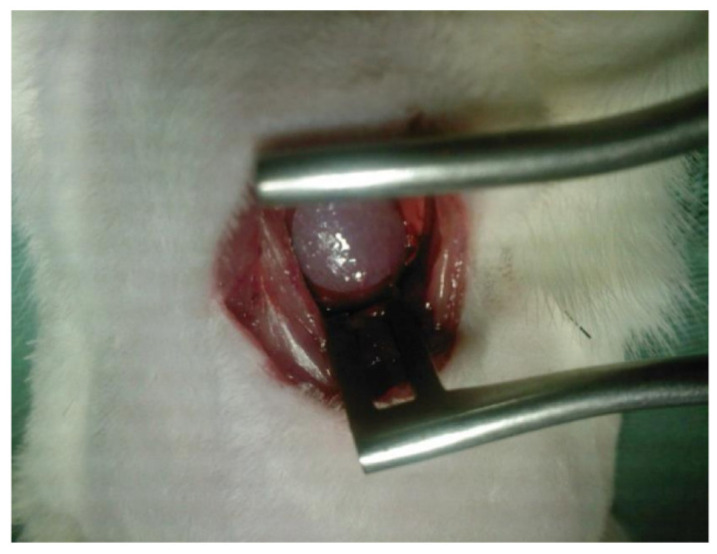
Placement of a cellulose patch on the left ventricle of a Wistar rat. This study aimed to use BC membrane patches containing cocultured cells to limit myocardial postinfarction pathology. Adapted from [159,165].

Meanwhile, patches made from BC membranes containing co-cultured cells (bone marrow and mesenchymal stem cells) were tested in vivo for their prospects as therapeutic patches targeting ischemia heart diseases. Results demonstrated that in comparison with BC membranes without cellular treatment, the patches with cells led to an improvement in a left ventricular ejection fraction, whereas those without cells were better in preserving cardiac dimensions [159]. Figure 8 shows placement of the BC scaffold on the left ventricle.

In another development, BC tubes were created in situ by cultivating strains of *Gluconacetobacter* in a special tube-like reservoir over a period of 7 days for each graft. The resultant tubes exhibited desirable mechanical and suturing attributes when tested in vivo over a period of 3 months. They were found to be suitable replacements for the carotid arteries of sheep following their ability to provide a suitable platform for the neoformation of a three-layered vascular wall [166].

#### 5.2.4. Corneal

The current growing concern worldwide is corneal vision loss due to damage to the cornea either by an object or by infection leading to a sight-threatening inflammatory condition called keratitis. Worldwide about 10 million people suffer from corneal vision loss every year [167]. The current therapeutic options and the corneal transplant approaches have their own limitations [167]. Hence, novel alternative routes are in high demand. The development of smart biomaterial-based tissue engineering approaches for the development of an artificial cornea has been progressing well for decades. The properties of BC such as optical transmittance, viscoelasticity, porosity, water holding capacity, and biocompatibility make it a promising candidate for a scaffold for corneal replacement [168]. There are quite a few studies where BC composites have been tested in the context of corneal tissue engineering.

The first report on the use of BC scaffolds for corneal tissue engineering was reported by Hui et al. in 2009 [169]. The results demonstrated that the BC scaffold supported the growth and proliferation of human corneal stromal cells. Jia et al. (2018) successfully developed hyaluronic acid/BC fibril-based composites by physical gelling with improved viscoelasticity, transmittance, and porosity due to the integration of BC nanofibrils. These mechanical properties were close to the mechanical properties of the cornea, thus concluding that this material has potential for application in artificial corneas [170,171]. In another study, BC impregnated with PVA hydrogel composites was tested for its thermo-mechanical properties, light transmittance, and water retention capability. The results showed that the composites exhibited suitable properties for artificial corneal replacement [172]. In 2020, Han et al. and his co-workers successfully developed a novel construct consisting of BC/poly(vinyl alcohol) (PVA) as a potential substitute for the corneal stroma. The properties exhibited by this novel construct such as optical transparency, water retention, morphology, surface functional group analysis, and porosity were improved significantly as compared to the neat BC hydrogel and thus could be suitable for corneal stroma engineering. The in vitro biocompatibility study and in vivo study on rabbits suggested that this novel material retains its integrity, stability, and transparency of the corneal stroma after intrastromal implantation more than BC alone. Thus BC/PVA composites have significant potential in corneal stromal engineering. However, dehydration is an issue with the material presumably due to the PVA content that needs to be solved before its further implementation [171].

**Figure 9:**
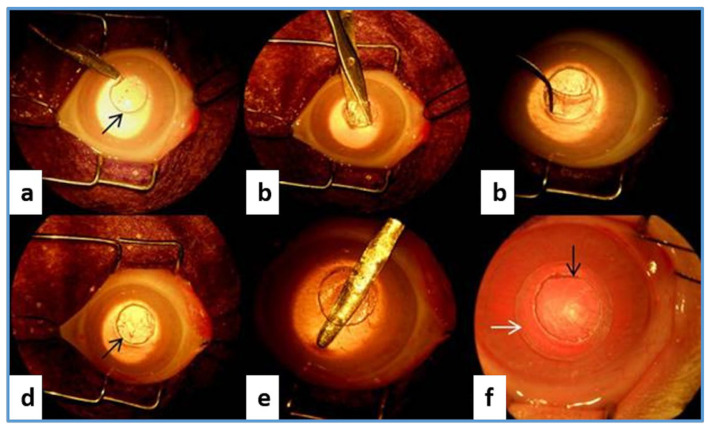
Implantation of BC/PCL and BC scaffolds in a rabbit’s cornea by a surgical technique (12× magnification). (**a**) Edge of the corneal trepanation (arrow). (**b**,**c**) Lamellar dissection. (**d**) Edge of complete superficial lamellar keratectomy (arrow). (**e**) Intrastromal insertion of spatula to produce a pocket. (**f**) Insertion of the membrane into the interlayer pocket; edge of corneal trepanation (black arrow); edge of the membrane inside the interlayer pocket (white arrow) (adapted from Sepulveda et al. [173]).

In an in vivo experiment by Sepulveda et al., BC and BC/polycaprolactone (PCL) scaffold membranes were tested on an artificially damaged rabbit’s cornea. The clinical observation revealed that the presence of BC and BC/PCL scaffold induced a moderate inflammatory process on the implanted site and impaired epithelial cell growth. Additionally, the scaffold led to the reduction of collagen production and generation of fibrous tissues, which led to the conclusion that the BC implants were not suitable as a corneal implant in rabbits [173]. Figure 9 demonstrates the implantation of BC/PCL and BC scaffolds in rabbit cornea by a surgical technique. In another study by Zang et al. [174], the biocompatibility of BC as a scaffold material was evaluated in in vitro and in vivo conditions on rabbit corneal epithelial and stromal cells. The results demonstrated 100% biocompatibility of the cells on BC, concluding that BC has potential as a scaffold in corneal tissue engineering which completely contradicted the observations made by Sepulveda et al. [173]. This uncertainty shows that there is clearly a need for further in-depth investigation about its realistic applications.

#### 5.2.5. Liver

**Figure 10:**
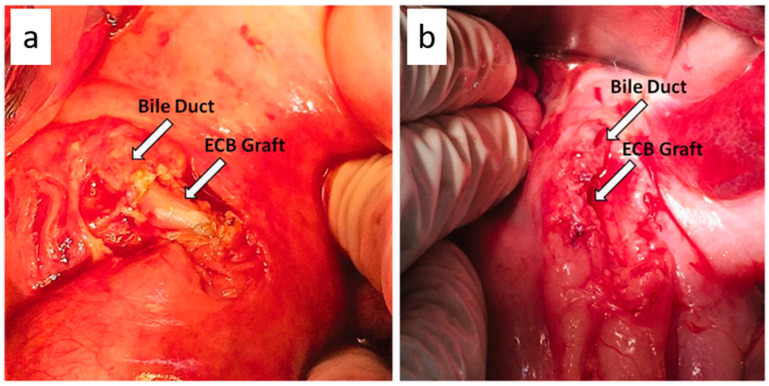
Longitudinal incisions on the anterior wall of the common bile duct and implanted cellulosic exopolysaccharide biopolymer (ECB); a BC film. The appearance of implanted BC films reoperated after 330 days (**a**) and 150 days (**b**). Reprinted with permission from reference [178], copyright © 2020, SAGE publication.

Liver is the largest internal organ of the human body, involved in more than 500 different functions that include regulation of glucose homeostasis and bile secretion, synthesis of lipids, lipoproteins, amino acids, proteins, vitamins, regulations of growth factors, and drugs/toxin metabolism [175]. Therefore, any damage to the liver can lead to serious life-threatening consequences. Fortunately, the liver has an innate capacity for restoration of its functions. However, this is not always the case; in certain clinical conditions, it fails to regenerate itself and therefore needs urgent treatment. The orthotropic liver transplant and extracorporeal devices are current strategies to combat such pathological conditions, but at the same time, they have their own limitations. Over 800 million people worldwide suffer from liver diseases leading to about 2 million deaths every year [176,177]. In this context, liver tissue engineering is thought to be a promising option in the future that could mimic the complex micro-architecture of the liver and provide a long-term solution by developing biomaterial-based tissue constructs. Previous studies have explored purified cellulose from plant origin in combination with hydrogels to successfully differentiate human liver cells; however, BC-based scaffolds in liver tissue engineering have not yet been fully explored. So far De Abreu et al. and co-workers [178], successfully used BC films for the repair of bile duct injury (10 mm longitudinal incision) in pig. The BC film demonstrated good biocompatibility by successfully treating a large elliptical defect in the bile duct and completion of the healing process after 330 days. It also restored biliary flow continuity and normal liver functions (Figure 10). The use of BC constructs in liver tissue engineering needs to be explored further.

#### 5.2.6. Skin

Being the first line of defence, the skin is undoubtedly one of the most vulnerable organs of the body. Although skin has the highest regenerative ability compared to other tissues, it cannot be completely repaired by itself, resulting in skin deformities. The current autologous skin transplantation (AST) treatment is commonly used as a treatment regime, but limitations such as lack of donors and post-transplantation complications limits the use of AST [179]. Biomaterial-based skin tissue engineering instead can provide concrete solutions for the treatment of skin disorders. BC has numerous advantageous qualities for skin tissue engineering; therefore, BC composites are being continuously explored for skin tissue engineering. Although, collagen and chitosan are the mostly commonly used biomaterials in wound dressing, and several commercial products with these polymers are already out in market, BC is also an FDA approved biomaterial due to presence of low levels of endotoxin (<20 EU per device) [30]. Therefore, there are several BC-based products for wound dressing that are already commercialised and available in the market. These include Biofill^®®^, Robin goad, London, England; XCell^®®^, Xylos Corporation, Langhorne, PA, USA; Bionext^®®^, Bennett Health, Texas, United States; Dermafill^TM^, Cellulose Solutions Ltd, Colquitt, Georgia; Gengiflex^®®^, Biofill Produtos Biotechnologicos Ltd, Curitiba, PR, Brazil; Cellulon^TM^, CP Kelco Atlanta, Georgia; and Membracel^®®^, Vuelo Pharma, Curitiba – PR Brazil [7,30]. However, despite extensive research in designing of BC-based scaffolds for skin tissue engineering, the goal of replacing skin autografts with the BC-based scaffolds remains challenging. However, considerable progress has been made in this direction in the recent years.

BC incorporated with keratin protein as a filler was produced by both in situ and post-production (ex situ) modification; the resultant composites successfully demonstrated improved adhesion, proliferation, and morphology of skin fibroblast cells (Detroit 562) and skin keratinocyte cells (HS2) [180]. In another study, Azarniya et al. and co-workers have improved cell adhesion and proliferation of fibroblast cells (L929) on a novel composite scaffold made up of BC/keratin nanofibres and tragacanth natural gum (TNG) [181]. In a separate study Azarniya et al. also demonstrated that graphene oxide (GO) nanosheets reinforced with chitosan/BC nanofibrous composites using electrospinning, exhibited an enhanced tensile strength and elastic modulus, and decreased elongation and hydrophilicity were observed as compared to pristine chitosan/BC nanofibrous composite. These results suggested that this novel composite could be potentially suitable for skin tissue engineering [182].

Sajjad et al. (2019) [183] designed a novel BC and montmorillonite (MMT) (BC/MMT) nanocomposite that combined the wound healing property of BC and antibacterial properties of MMT. The enhanced wound healing, tissue regeneration, regenerative epithelialisation, vascularisation, and healthy granulation was observed when tested on mice wounds. In fact, more recently the BC-hydrogel (BCH) was incorporated with MMT by the deposition method and tested on the skin wound model in mice after pressure injury. The results suggested that the modified BCH-MMT composite induced cutaneous healing by reducing lesion area and inflammation, subsequently promoting wound re-epithelialisation [184]. The most recent study by Oran et al. (2022) [185] reported that the BC-composite scaffold fabricated with quince seed mucilage (a glucuronoxylan polysaccharide hydrogel) resulted in modified and increased swelling behaviour of the composites supported enhanced fibroblast proliferation and adhesion. Meng et al. and co-workers [186] synthesised BC immobilised with resveratrol (RSV); the resultant scaffold retained normal collagen-bundling pattern and induced re-epithelialisation in defective rat epidermis. The improved crosslinking of the BC obtained by addition of Ti^4+^ during in situ cultivation induced remarkable mechanical properties [187], demonstrating that these modified BC properties could be suitable for use in skin tissue engineering.

**Figure 11:**
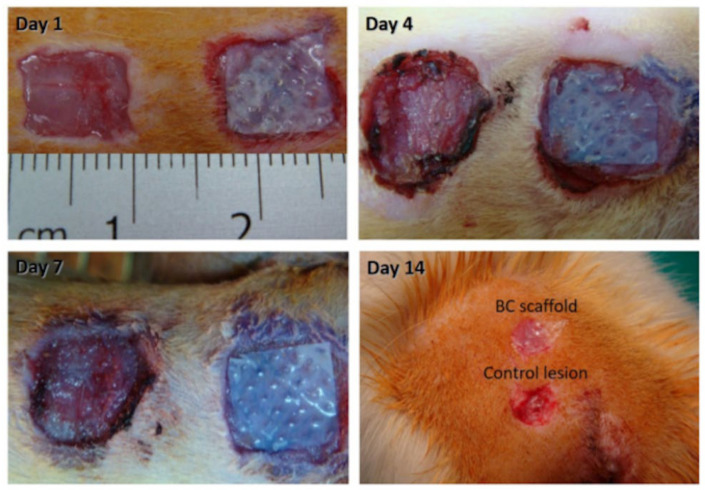
Rat model of skin defects healing over time without (**left side**) or with bacterial cellulose scaffold covering (**right side**) (Adapted from Cheng et al. [188]).

Cheng et al. [188] evaluated the biocompatibility of the BC scaffold on human adipose stem cells (hASCs) in vitro and in vivo using a rat skin model, as shown in Figure 11. The results indicated that the BC scaffold supported epithelial regeneration, wound healing, normal stemness function of cells, promoted keratinocyte differentiation, improved skin extracellular matrix deposition, and controlled excessive inflammation, demonstrating that it could be a promising product for skin injury repair.

Altun et al. [189] have produced a BC/poly(methylmethacrylate) (PMMA) fibre bandage using the pressurised gyration method. The in vitro study of the resultant BC/PMMA scaffold on Saos-2 cell line showed increased biocompatibility. BC reinforced with sodium alginate cross-linked with Ca^2+^ improved the swelling and thermal and mechanical properties of the resultant BC composite as compared to neat BC [190].

In medical applications, BC was first used in wound dressings for improved tissue regeneration [191]. The main criteria for successful wound dressings are to provide a moist environment, inhibit bacterial infections, be non-toxic and non-allergenic, promote heat insulation, and allow easy transportation of gases [9]. BC can provide reduced pain, acceleration of healing, and a great fit to the body, making it suitable for skin care applications [191]. There were several in vivo and in vitro studies that have been conducted on novel BC-containing composites in attempts to provide an ideal micro-environment for wound healing. In addition, many studies have tested whether incorporating drugs into BC can give BC antimicrobial properties needed for wound dressing applications. Das et al. synthesised a wound dressing material via the impregnation of polycaprolactone (PCL) into the BC matrix, which was further functionalised with gentamicin (GEN) and streptomycin (SM). In vitro studies showed excellent antimicrobial activity against *E.coli* and *S. aureus* [192]. Chuah et al. (2018) used a similar method and synthesised a BC-Poly(acrylic acid) hybrid hydrogel with grafted amoxicillin. This hydrogel composite was able to release drugs with controllable antimicrobial ability. The additional hygroscopicity analysis also revealed increased mechanical stability of the scaffold over BC alone [193]. Other studies have used silver to incorporate the antibacterial properties needed for controlling infection. Tabaii et al. fabricated antimicrobial silver nanoparticle-containing membranes using BC, (AgNP)/BC. The resultant membranes showed a controlled release of silver ions (Ag+), good swelling, excellent microbial resistance towards *E. coli* and *S. aureus*, and good biocompatibility on peripheral blood mononuclear cells [194]. Gupta et al. and Wu et al., also found similar positive antimicrobial activity when employing AgNPs and BC [195,196]. Silver nanowires used by Wan et al. have also shown positive results. Their structure showed sustained Ag+ release rates, greater proliferation of keratinocytes cells, and improved skin regeneration when tested in vivo compared to pure BC [197].

Hamedi et al. (2021), synthesised amine-functionalised BC hydrogel with schizophyllan (SPG) with zinc oxide (ZnO) nanoparticles. The resultant composite showed an increased swelling degree, higher tensile strength, and remarkable antibacterial properties when compared to pure BC. It was also found that this hydrogel could stimulate the proliferation of human fibroblast cells with no toxic effects [198]. Fu et al. (2021) and Wichai et al. (2019), both employed chitosan in BC composites to add antibacterial properties. Fu et al. used chitosan (CS)/oxidised BC composites, whilst Wichai et al. fabricated a BC composite containing sodium alginate (AG), CS, and copper sulfate (Cu). Further study showed both composites were able to inhibit *E.coli* and *S. aureus* growth, with no cytotoxicity in vitro towards human dermal fibroblast cells and also proved biocompatible in mice, in vivo [59,199].

Orlando et al. developed functionalised antibacterial BC patches by a heterogeneous reaction with two active epoxides, namely glycidyl trimethylammonium chloride and glycidyl hexadecyl ether. The modified BC patches showed 53% and 43% reduction of cell numbers when tested against *E. coli* ATCC 8739™ and *S. aureus* subsp. Aureus Rosenbach 6538P™, respectively. Moreover, both modified and unmodified BC showed excellent biocompatibility (90–100% cell viability) for keratinocytes (HaCaT cells), hence representing a promising material for wound dressing applications [200].

## 6. Conclusions and Future Perspectives

The cost associated with BC production and low productivity are the two main issues that are currently hindering the wider commercial applications of BC. Therefore, it is proposed that the use of cheap agro-industrial waste as a substrate instead of using traditional sugars could be the promising alternative to produce BC in a sustainable manner for economic, environmental, and social benefit. The designing of novel bioreactors such as rotary disc reactors (RDR), optimisation of process parameters, and use of high-yielding microbial strains are recommended to improve the overall production efficiency. Additionally, advanced metabolic engineering approaches can significantly help reprogramming of the microbial strains bringing new prospects to improve BC production.

Although BC scaffolds certainly added value to the tissue engineering field and have made considerable progress over the last decade, current research needs further progression. Therefore, the use of BC-based scaffolds for practical applications is far from reality. The key challenges with BC-based scaffolds are irregular porosity and non-biodegradability in the human body.

Another key challenge is that functionalised BC scaffolds have to match the properties of the native ECM in order to support cell growth and tissue development. Several studies used different additives to introduce distinct properties to the BC scaffolds including nanoparticles (silver derivatives, gold, hydroxyapatite, and graphene) and other biocompatible polymers (chitosan, alginate, and gelatin). There are several examples of BC-derived scaffolds that have improved mechanical and structural properties post-modification and exhibited remarkable biocompatibility with various tissues both in vivo and in vitro. For example, bacterial cellulose/chitosan/nanohydroxyapatite (BC/CS/nHA), bacterial cellulose/sodium alginate (BC/SA), and alginate/bacterial cellulose nanocrystals/chitosan/gelatin (Alg/BCNs/CS/GT) improved adhesion, proliferation, and regeneration of bone tissue and BC/decellularised cartilage extracellular matrix (BC/DCECM) scaffolds achieved neocartilage tissue regeneration. Similarly, in soft tissue engineering, there are several BC-based scaffolds that exhibited promising results including BC/carboxymethyl cellulose/hydroxyapatite (BC/CMC/HA) with kidney cells; conductive scaffold BC/poly(3,4-ethylene dioxythiophene)-sulphonated nanofibres (BC/PSN) and nerve growth factor/chitosan nanoparticles/oxidised BC (OBCNGF/CSNPs/OBC) scaffold with neural cells; poly(2-hydroxyethyl methacrylate) (PHEMA/BC) and 3D printed TEMPO-modified nanocrystalline cellulose/methacrylated gelatin (mNCC/MeGel (mNG)) with cardiac cells; BC/Poly(vinyl alcohol) (BC/PVA) and BC/polycaprolactone (BC/PCL) with corneal cells; and BC/keratin/tragacanth natural gum (TNG), BC hydrogel/montmorillonite (BCH/MMT) and BC/poly(methylmethacrylate) (PMMA) with skin cells.

However, further research is needed to overcome the problems associated with biodegradability, 3D structure instability after chemical modifications, and inadequate porosity (e.g., small pore size) of the BC scaffolds. The BC scaffolds with hydrogels and PVA, hydroxyapatite, and gelatin can help improve the porosity of the BC scaffolds significantly. The biodegradability of the BC scaffolds can be improved with oxidation. Advanced 3D printing techniques are recommended to produce precise and functionalised BC scaffolds for various tissue engineering applications. Hence, overall there is a large research space to improve BC-based composites and blends as highly sophisticated materials for tissue engineering applications.

## Figures and Tables

**Table 1:** Examples of potential BC composites and blends tested for bone tissue engineering.

Composite/Blend	Properties	Reference
BC/gelatin/hydroxyapatite	Supported good adhesion and great cell proliferation and differentiation in mesenchymal stem cells derived from rat bone marrow.	Ran et al. [122]
BC mineralised with nano hydroxyapatite (nHA)	Human-derived bone marrow stem cells adhered, proliferated, and differentiated on this BC composite.	Favi et al. [52]
BC modified with gel and hydroxyapatite (HAp)-coating	Showed improved adhesion, viability, differentiation, and proliferation of human-derived bone marrow stem cells, compared to composite of BC mineralised with nano hydroxyapatite.	Huang et al. [123]
BC/poly(ethylene glycol) composite	Improved cell viability, adhesion, and proliferation of 3T3 fibroblast cells.	Wu, et al. [124]
BC/gelatin scaffold loaded with VEGF-silk fibroin nanoparticles	This scaffold was seen to significantly promote vascularisation after implantation into the defective bone.	Wang et al. [125]
BC/silk fibroin sponge scaffold	This composite scaffold showed no cytotoxicity or genotoxicity against L929 and V79 cells. Also showed great cell adhesion.	Barud et al. [126]

## Data Availability

Not applicable.
